# Delafloxacin suppresses DEPDC1 expression and induces G2/M arrest in non‐small cell lung cancer cells: A drug repurposing study

**DOI:** 10.1002/smo2.70050

**Published:** 2026-05-28

**Authors:** Noman Ali, Farishta Shafiq, Mishal Iftikhar, Muhammad Shahzad Zafar, Muhammad Shoaib

**Affiliations:** ^1^ School of Bioengineering Dalian University of Technology Dalian China; ^2^ State Key Laboratory of Fine Chemicals School of Chemical Engineering Dalian University of Technology Dalian China; ^3^ Institute of Advanced Study Shenzhen University Shenzhen China; ^4^ Department of General Surgery Shenzhen University General Hospital Institute of Precision Diagnosis and Treatment of Digestive System Tumors Shenzhen China; ^5^ SusMat‐RC Mohammed VI Polytechnic University Ben Guerir Morocco

**Keywords:** ADMET analysis, DEPDC1, lung dose‐dependent suppression, molecular docking

## Abstract

Emerging evidence highlights the oncogenic role of the Dishevelled, Egl‐10, and Pleckstrin domains containing 1 (DEPDC1) gene in lung cancer progression, making it an attractive therapeutic target. In this study, we investigated the anti‐cancer potential of Delafloxacin, a fluoroquinolone antibiotic, in DEPDC1‐overexpressing non‐small cell lung cancer, NCI‐H1299 cells. In silico docking of 1399 FDA‐approved compounds using AutoDock Vina confirmed the strong binding affinity of Delafloxacin to the DEPDC1 active site (binding energy −7.1 kcal/mol). Delafloxacin treatment resulted in dose‐dependent suppression of cell proliferation, with an IC_50_ of approximately 30 μg/mL. RT‐qPCR revealed significant downregulation of DEPDC1 and several key regulators of proliferation (RAS, EGFR), mitotic progression (Cyclin‐dependent kinase 1 (CDK1), CCNB2, KIF2C), and survival (BIRC5). Cell cycle analysis demonstrated G2/M phase accumulation upon Delafloxacin treatment, consistent with transcriptional repression of CDK1 and CCNB2. In vivo, Delafloxacin administration (15 mg/kg) significantly reduced tumor growth in a xenograft mouse model without overt toxicity. While these findings identify Delafloxacin as a candidate for repurposing in lung cancer, the observed downregulation of DEPDC1 represents a correlative finding rather than validated direct targeting. This study provides a rationale for further investigation of Delafloxacin in DEPDC1‐associated malignancies.

## Graphical abstract

This study presents Delafloxacin repurposed as a DEPDC1‐targeting therapeutic aiming to suppress lung cancer progression. It highlights a novel strategy for lung cancer treatment with potential clinical applications.

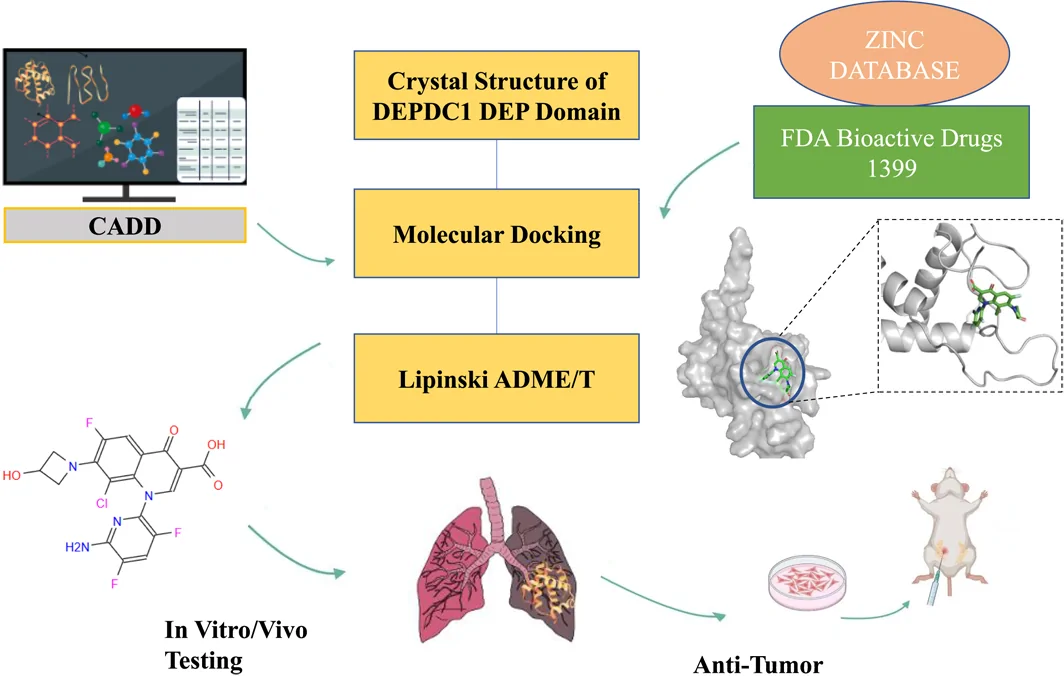

## 1 INTRODUCTION

Cancer refers to a group of multiple diseases with common characteristics. It is characterized by unregulated growth and spreading to other body parts.^[1]^ Lung cancer is one of the most common types, and its mortality rate is increasing year after year. The diagnosis of this disease is usually made in the late stage. But patients with lung cancer suffer more than those with other cancer types, even in earlier stages. During stage I of lung cancer, the survival rate is mostly below 70%. The survival rate for breast cancer in stage I is almost 95%.[^2^, ^3^] According to the report of the American Cancer Society, in 2017, in the USA, nearly 40,000 lung cancer cases were reported, of which 28,000 patients died, approximately.^[4]^ The reason for the high mortality rate is the resistance to drugs and distant metastasis.^[5]^ DEPDC1 is the DEP domain‐containing 1 gene (Dishevelled, Egl‐10, and Pleckstrin). Its location is 1p31.3. This gene is conserved among many species, from C. *elegans* to humans.^[6]^ The highly conserved 92‐kDa protein, encoded by DEPDC1, plays an essential role in various biological processes, including cell cycle progression, cell proliferation, signaling transduction, and apoptosis.^[7]^

The DEPDC1 gene plays a crucial role in various cancers, including hepatocellular carcinoma (HCC),^[8]^ bladder cancer,^[9]^ breast cancer,^[10]^ and prostate cancer.^[11]^ Katagiri and colleagues reported that in bladder tumors, DEPDC1 was upregulated, but it was still undetectable in 24 different normal tissues, except the testis,^[9]^ whereas in normal tissues, its role is still unknown. According to Guo et al., DEPDC1 may increase the expression of chemokine receptor 6 and chemokine ligand 20 (CCL20) in hepatocellular carcinoma cells, thereby promoting cell invasion and proliferation via the CCL20/CCR6 signaling axis.^[12]^ DEPDC1 has emerged as a key regulatory gene in lung adenocarcinoma (LUAD), with elevated expression levels identified by weighted gene co‐expression network analysis and protein–protein interaction mapping. Clinical data indicate that patients with higher DEPDC1 expression tend to have poorer overall survival outcomes. Further gene set enrichment analysis suggests that DEPDC1 contributes to tumor progression,^[13]^ primarily by activating NF‐κB signaling pathways,^[14]^ which regulate critical processes such as cell cycle progression and epithelial–mesenchymal transition. Experimental suppression of DEPDC1 has been shown to significantly reduce both the proliferative and migratory capacity of lung cancer cells, highlighting its potential as a therapeutic target.^[15]^ DEPDC1 may contribute to non‐small cell lung cancer (NSCLC) tumorigenesis and can be applied as a biomarker for diagnosis.^[16]^ One of the essential mechanisms underlying biological activity is the interaction between biomolecules, which drives metabolic regulation and cellular signaling. These molecular interactions are foundational to the maintenance of life.^[17]^ In modern biomedical research, computational simulations such as molecular docking have emerged as indispensable tools for identifying and modulating bioactive compounds.[^18^, ^19^, ^20^] Molecular docking enables the prediction of binding interactions between a small molecule and a target protein at the atomic level, facilitating the identification of potential therapeutic candidates.^[21]^ This structure‐based approach relies on high‐resolution 3D protein models obtained through X‐ray crystallography, cryo‐electron microscopy, or NMR spectroscopy.^[22]^.

In drug discovery, molecular docking provides a cost‐effective, high‐throughput approach for screening large compound libraries for potential inhibitors.[^23^, ^24^] In this study, AutoDock Vina was used to screen 1399 FDA‐approved drugs and bioactive compounds for binding to DEPDC1, a cancer‐testis antigen increasingly implicated in various malignancies. Among the top candidates, Delafloxacin exhibited the strongest binding affinity (−7.1 kcal/mol), indicating a likely interaction with the DEPDC1 active site. DEPDC1, a conserved gene implicated in cell cycle regulation, apoptosis, and oncogenic signaling,[^25^, ^26^] was significantly downregulated the following Delafloxacin treatment. This downregulation corresponded with the repression of downstream effectors, including Cyclin‐dependent kinase 1 (CDK1),^[27]^ CCNB2,^[28]^ and KIF2C^[29]^, leading to G2/M cell cycle arrest in NCI‐H1299 cells. Flow cytometric analysis further validated the accumulation of cells in the G2/M phase, supporting the hypothesis that Delafloxacin disrupts mitotic progression by inhibiting DEPDC1.^[30]^ These findings establish a compelling link between computational predictions and biological outcomes, positioning Delafloxacin as a promising therapeutic agent targeting DEPDC1‐driven oncogenesis.

## 2 MATERIALS AND METHODS

### 2.1 Materials

ZINC database (https://zinc.docking.org/substances/subsets/), Intel® Xeon® E5‐1603 0 @ 2.80 GHz quad‐core processor, Ubuntu Operating System, AutoDock Vina, NCI‐H1299 NSCLC, RPI‐1640 (Gibco), Trypsin‐EDTA (Gibco), phosphate‐buffered saline (PBS) (Solarbio), fetal bovine serum (FBS) (Sigma), Penicillin‐Streptomycin (Gibco), CCK‐8 kit (Dojindo), Trizol (Thermofisher), propidium iodide (PI) (Biorad), FACS (Biorad).

### 2.2 Molecular docking of DEPDC1 with FDA‐Approved drugs and bioactive compounds from the ZINC database

This study aimed to evaluate the interaction of DEPDC1 (PDB ID: 2YSR) with a subset of 1399 FDA‐approved drugs and bioactive compounds sourced from the ZINC database (https://zinc.docking.org/substances/subsets/), which are known for their therapeutic potential. All computational procedures were performed on a workstation running the Ubuntu operating system, powered by an Intel® Xeon® E5‐1603 0 @ 2.80 GHz quad‐core processor.

Protein‐ligand docking was performed using AutoDock Vina,^[31]^ which is known for its high‐scoring functions, multithreading capabilities, and efficient optimization strategies. The three‐dimensional (3D) structure of the DEPDC1 protein (2YSR.pdb) from the Protein Data Bank was pre‐processed through homology modeling and structure refinement. These included removing water molecules, adding hydrogens (3D protonation), and minimizing energy using the MMFF94X force field with solvation parameters and chiral geometry constraints (gradient threshold: 0.05). The docking grid was centered on the predicted DEPDC1 binding pocket with the following center coordinates: *x* = 0.268, *y* = −3.997, and *z* = 0.043. The grid box dimensions were set to 44 Å × 40 Å × 40 Å (size_*x* = 44, size_*y* = 40, size_*z* = 40), ensuring complete coverage of the active site cavity. A grid spacing of 0.375 Å was applied. The exhaustiveness parameter was set to 10 to ensure sufficient conformational sampling, while the number of output binding modes (num_modes) was set to 10 with an energy range of 3 kcal/mol. These parameters were selected to balance computational efficiency with reliable sampling of ligand–protein interactions.

The ligand structures were retrieved from the ZINC database in 3D format and prepared for docking. AutoDock Vina was run using optimized configuration to perform docking simulations, predicting each compound's optimal binding poses and associated binding affinities. Post‐docking analysis involved visualizing and interpreting ligand‐protein interactions, including hydrogen bonding and hydrophobic contacts, using UCSF Chimera and PyMOL software packages.

In the docking simulations, the DEPDC1 structure was treated as a rigid receptor, while ligand flexibility was permitted through rotation of torsional bonds. No explicit side‐chain flexibility of the protein was modeled. Crystallographic water molecules were removed during protein preparation, and polar hydrogens were added prior to docking. Solvation effects were implicitly accounted for by the empirical scoring function implemented in AutoDock Vina, which incorporates terms representing hydrophobic interactions and hydrogen bonding without explicit solvent representation. Although this rigid docking approach does not capture full protein conformational dynamics or explicit solvent effects, it is widely employed in high‐throughput virtual screening and provides reliable comparative estimates of ligand–protein binding affinity.

### 2.3 Prediction of ADMET and bioactivity properties

Following molecular docking, the top‐ranking ligands based on binding affinity were selected for further analysis of pharmacokinetic and bioactivity characteristics. These compounds were evaluated using the Molinspiration Cheminformatics platform (http://www.molinspiration.com/), which computes key ADME (Absorption, Distribution, Metabolism, and Excretion) properties. Drug‐likeness was assessed using Lipinski's Rule of Five (RO5), which requires molecular weight ≤ 500 Da, ≤ 5 hydrogen bond donors, ≤ 10 hydrogen bond acceptors, and log*P* ≤ 5. These criteria help in the drugability of the compounds.

### 2.4 Cell culture

NCI‐H1299 lung cancer cells were used in this study; NCI‐H1299 cells were selected based on published reports demonstrating elevated DEPDC1 expression in this line, making it a suitable model for initial evaluation of DEPDC1‐associated cellular responses as NCI‐H1299 has been shown to exhibit higher DEPDC1 expression. NCI‐H1299 human non‐small cell lung carcinoma cells were cultured according to ATCC guidelines. The complete medium consisted of RPMI‐1640 supplemented with 10% FBS, 100 U/mL penicillin, and 100 μg/mL streptomycin. The cells were maintained at 37°C in a humidified atmosphere with 5% CO_2_, and the medium was replaced every 2–3 days. Subculturing was performed when the cells reached approximately 70%–80% confluency. Cells were washed with PBS, detached using 0.25% trypsin‐EDTA, and passaged at a recommended split ratio of 1:3 to 1:6, depending on growth requirements. For all subsequent experiments, cells had less than 10 passages.

### 2.5 IC_50_ by cell counting kit‐8 (CCK‐8) assay

For drug treatment experiments, NCI‐H1299 cells were seeded in 96‐well plates at a density of 10,000 cells per well in 100 μL of complete growth medium and incubated overnight to allow cell attachment. The following compounds were tested: Delafloxacin, Fujiglucon, and Afatinib. Each drug was prepared in complete‐growth medium at final concentrations of 1, 2, 4, 8, 16, 32, 64, and 132 μg/mL. Control wells received an equivalent volume of DMSO.

Cells were treated for 48 h at 37°C in a humidified incubator with 5% CO_2_. Following treatment, cell viability was assessed using the Cell Counting Kit‐8 (CCK‐8; Dojindo) according to the manufacturer's instructions. Briefly, 10 μL of CCK‐8 solution was added to each well, and the plates were incubated for 2 h at 37°C. Absorbance was measured at 450 nm using a microplate reader (insert model and manufacturer, if applicable).

The percentage of cell viability was calculated using the formula:

$$\text{cell}\;\text{viability}\left(\%\right)=\frac{A_{\text{treatment}}-A_{\text{blank}}}{A_{\text{Control}}-A_{\text{blank}}}\times 100$$

where $A_{\text{treatment}}$ is the absorbance of the drug‐treated well, $A_{\text{control}}$ is the absorbance of vehicle‐treated control wells, and $A_{\text{blank}}$ is the absorbance of wells containing medium and CCK‐8 without cells.

All experiments were performed in triplicate and independently repeated at least three times to ensure reproducibility. Data were analyzed and graphed using GraphPad Prism software (version 8, GraphPad Software).

### 2.6 Gene expression analysis via RT‐qPCR

NCI‐H1299 human non‐small cell lung carcinoma cells were cultured in RPMI‐1640 medium supplemented with 10% FBS and 1% penicillin–streptomycin at 37°C in a 5% CO_2_ atmosphere. Cells were seeded in 6‐well plates and allowed to adhere overnight. The next day, cells were treated with Delafloxacin at concentrations of 0 (control), 10, 20, and 40 μg/mL for 24 h.

Total RNA was extracted using TRIzol reagent according to the manufacturer's protocol. cDNA synthesis was performed using a reverse transcription kit. RT‐qPCR was conducted employing SYBR Green chemistry on a real‐time PCR detection system (Table 1). Expression levels of DEPDC1, RAS, EGFR, KIF2C, CDK1, CCNB2, MAPK1, NID1, FN1, BIRC5, and TNFSF12 were measured. GAPDH served as the internal reference gene. Relative expression was calculated using the ΔΔCt method, and the results were plotted as fold changes relative to the control.

**TABLE 1:** Primer sequences designed for RT‐qPCR validation of gene expression.

No.	Gene	Forward primer	Reverse primer
1	DEPDC1	CTCGTAGAACTCCTAAAAGGCATG	CAACATCTTCCTGGCTTAGTTCTC
2	RAS	ACGCACTGTGGAATCTCGGCAG	TCACGCACCAACGTGTAGAAGG
3	EGFR	AACACCCTGGTCTGGAAGTACG	TCGTTGGACAGCCTTCAAGACC
4	KIF2C	TTCGCATCACGGCTCAGGAGAA	GGACTTGCTCTTCCATCTCCTC
5	CDK1	CATGGACCTCAAGAAGTACCTGG	CAAGTCTCTGTGAAGAACTCGCC
6	CCNB2	CAACCAGAGCAGCACAAGTAGC	GGAGCCAACTTTTCCATCTGTAC
7	MAPK1	ACACCAACCTCTCGTACATCGG	TGGCAGTAGGTCTGGTGCTCAA
8	NID1	ACATTGAGCCCTACACGGAGCT	GCCACTGGTAAGTGTAGATGCG
9	FN1	ACAACACCGAGGTGACTGAGAC	GGACACAACGATGCTTCCTGAG
10	BIRC5	CCACTGAGAACGAGCCAGACTT	GTATTACAGGCGTAAGCCACCG
11	TNFSF12	CGGAAAAGGAGAGCAGTGCTCA	TGGTTGCCACATCACCTCTGTC

### 2.7 Cell cycle analysis by flow cytometry

NCI‐H1299 cells (human non‐small cell lung carcinoma line) were cultured in RPMI‐1640 medium supplemented with 10% FBS and 1% penicillin‐streptomycin, maintained at 37°C with 5% CO_2_. Cells were seeded at 60%–70% confluence in 6‐well plates and treated with Delafloxacin at 0 (control), 10 μg/mL, or 20 μg/mL for 24 h. Treatments were performed in biological triplicates.

Following treatment, the cells were harvested, washed with PBS, and fixed in 70% ethanol at −20°C overnight. Fixed cells were stained with PI solution containing RNase A for 30 min at room temperature in the dark. DNA content was analyzed using a flow cytometer (e.g., BD FACSCalibur), and cell cycle phases were quantified using the Watson model‐based deconvolution in FlowJo software.

### 2.8 Effect of delafoxacin on NCI‐H1299 cells in vivo

*Animal care*: All animal procedures were performed in accordance with the Guidelines for care of Laboratory at Institute of Precision Diagnosis and Treatment of Digestive System Tumors Shenzhen University and were approved by the Institutional Animal Care and Use Committee (IACUC). Animals were housed under standard conditions with controlled temperature and humidity, a 12 h light/dark cycle, and ad libitum access to food and water.

*In vivo model*: Female BALB/c nude mice (4–6 weeks old; ∼18–22 g body weight) were housed under specific pathogen‐free (SPF) conditions with ad libitum access to food and water. For tumor xenograft establishment, 1 × 10^5^ NCI‐H1299 cells in 100 μL of PBS/Matrigel (1:1) were implanted subcutaneously into the right flank of each mouse. Tumor growth was monitored every 3 days using calipers, and tumor volume was calculated according to the formula:

$$\text{Tumor}\;\text{volume}\left({\text{mm}}^{3}\right)=\frac{\text{length}\times {\left(\text{width}\right)}^{2}}{2}$$

When tumors reached approximately 175 mm^3^, animals were randomized into two groups (*n* = 5 per group): a vehicle control group and a Delafloxacin‐treated group.

Delafloxacin was administered intravenously at a dose of 15 mg/kg every 3 days for 42 days. The drug was prepared in sterile saline (0.9% NaCl) at a concentration suitable to deliver the required dose based on the body weight of the animals. Throughout the treatment period, tumor volume and animal body weight were measured every 3 days to monitor therapeutic response and systemic toxicity. At the end of the experiment (day 42), animals were euthanized by CO_2_ asphyxiation, and tumors were excised, weighed, and documented.

### 2.9 Statistical analysis

All qantitative data are presented as mean ± standard deviation (SD) unless otherwise specified. All experiments were performed in at least three independent biological replicates (*n* ≥ 3), with technical triplicates for each condition in in vitro assays. IC_50_ values were calculated by nonlinear regression using a four‐parameter sigmoidal dose‐response curve (variable slope) model in GraphPad Prism 8.0. Goodness‐of‐fit was assessed by *R* ^2^ values (all > 0.95). Relative gene expression was calculated using the ΔΔCt method with GAPDH as the endogenous reference. Statistical significance was defined as *p < 0.05, **p < 0.01, ***p < 0.001, ****p < 0.0001. All statistical analyses were performed using GraphPad Prism version 8.0 (GraphPad Software, San Diego, CA).

## 3 RESULTS AND DISCUSSION

Lung cancer is one of the leading cancers worldwide, and finding new markers and therapeutic strategies is in high demand. The current study focuses on identifying an inhibitor of DEPDC1, an essential target in lung cancer associated with G2/M progression and tumorigenesis.

### 3.1 Structural characterization of the DEPDC1 binding pocket

To validate DEPDC1 as a molecular target, the three‐dimensional structure of its DEP domain (PDB ID: 2YSR) was thoroughly analyzed. As shown in (Figure 1), the structural surface (a) and ribbon (b) representations highlight the spatial distribution of residues forming the ligand‐binding pocket. These include aromatic residues (e.g., TRP, PHE, MET), polar residues (e.g., ARG, ASN), and hydrophobic residues (e.g., VAL, LEU, ALA), which collectively create a chemically diverse and accessible binding cavity. The protein structure was pre‐processed through energy minimization using the MMFF94X force field, with solvation and chiral constraints applied to ensure native‐like geometry. This refined structural model provided a high‐fidelity template for molecular docking simulations, enabling accurate prediction of ligand interactions within the DEPDC1 binding pocket. The identified residues corresponded well with key contact points observed in docking studies with Delafloxacin, Riboflavin, and Afatinib, confirming the functional relevance of the selected binding site.

**FIGURE 1:**
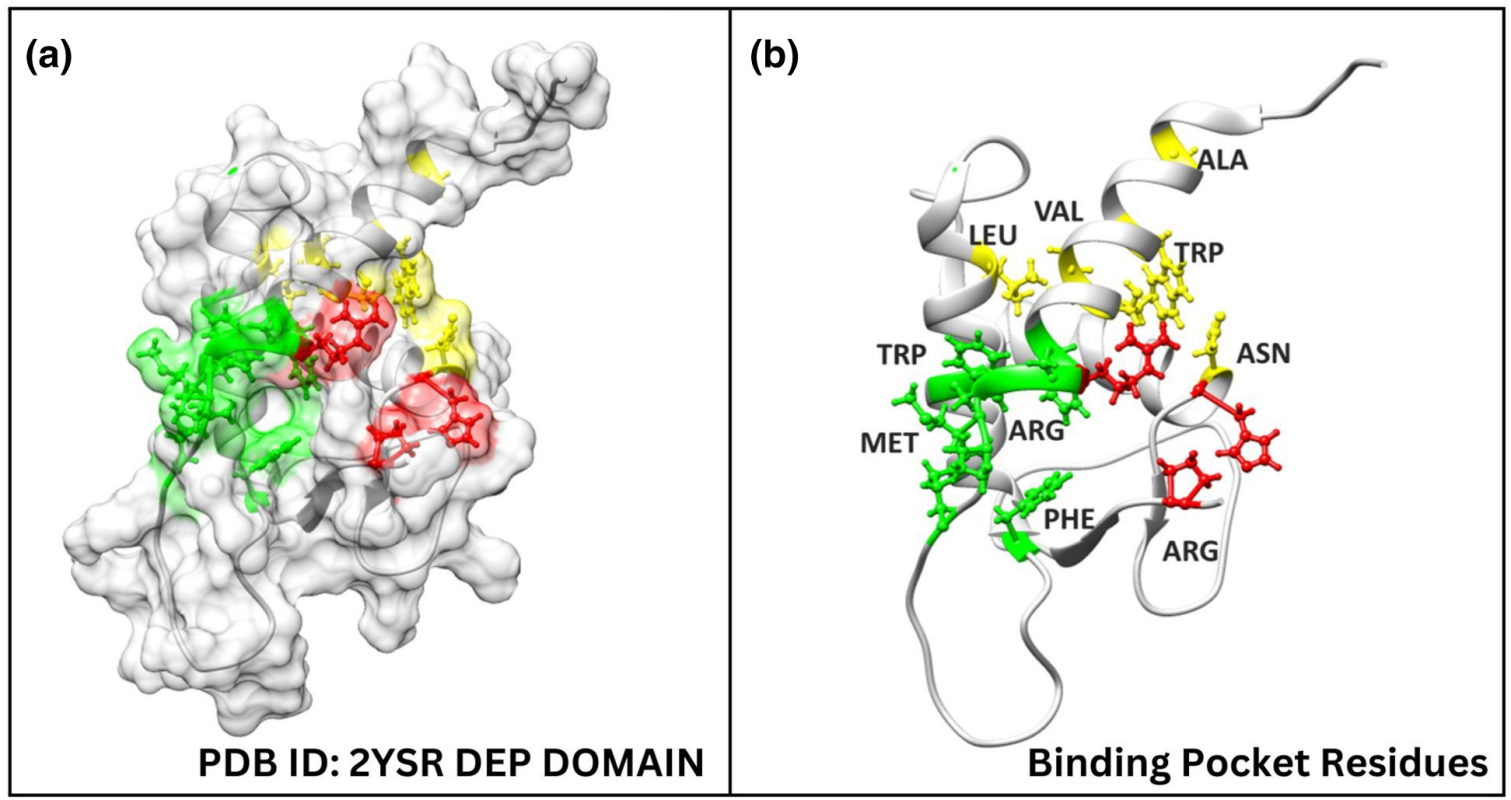
Binding domain and binding pocket residue of DEPDC1. (a), DEPCD1 domain retrieved from PDB. 1 (b), binding pocket of DEPDC1 showing binding residues.

### 3.2 Virtual screening and molecular docking analysis

A comprehensive in silico screening was performed on a library of 1399 FDA‐approved bioactive compounds using AutoDock Vina to identify potential inhibitors targeting the DEP domain of DEPDC1. The docking simulations generated binding affinity scores expressed in kcal/mol, which served as indicators of the strength and stability of the interaction between each compound and the target protein. Lower (more negative) binding energy values indicate stronger binding affinity and thus greater potential inhibitory activity. From the entire compound set, the top 15 ligands exhibiting the most favorable binding affinities, ranging from −5.0 to −7.2 kcal/mol, were shortlisted for further evaluation and detailed analysis (Table 2). This prioritized list of candidate molecules was the foundation for subsequent in vitro and in vivo evaluations to validate their therapeutic efficacy against DEPDC1‐driven lung cancer. The strong binding affinities identified through molecular docking provide a preliminary but robust framework for drug repurposing efforts aimed at accelerating cancer drug discovery.

**TABLE 2:** Docking results of 15 selected FDA‐approved ligands from the ZINC database.

No.	ZINC DB	Ligand name	Binding affinity (kcal/mol)
**1**	**ZINC000003827556**	**Delafloxacin**	−**7.1**
2	ZINC000003830944	IOHEXOL	−5.5
3	ZINC000002539702	Fujiglucon	−5
4	ZINC000001883067	Trinitrophenol	−5.5
5	ZINC000003813061	Bumetanide	−6.1
6	ZINC000000020220	Ciprofloxacin	−6.7
7	ZINC000000592419	Daxas	−6
8	ZINC000003918138	Zanamivir	−5.5
**9**	**ZINC000003976838**	**Afatinib**	*−* ***6***. ***8***
10	ZINC000001530922	Fosphenytoin	−6.3
11	ZINC000003842753	Viroptic	−6.6
12	ZINC000003826253	Moxifloxacin	−6.8
13	ZINC000004038341	Cleocin	−5.8
**14**	**ZINC000002036848**	**Riboflavin**	−**7.2**
15	ZINC000001035331	Ribasphere	−6.2

### 3.3 Top candidate selection and binding interaction analysis

Among the top 15 compounds identified through docking (Table 2), Riboflavin exhibited the highest binding affinity (−7.2 kcal/mol), followed closely by Delafloxacin (−7.1 kcal/mol) and Afatinib (−6.8 kcal/mol). While Riboflavin showed the strongest interaction energy, its poor drug‐likeness limits its therapeutic utility. In contrast, Delafloxacin and Afatinib not only demonstrated high binding affinities but also satisfied key drug‐likeness criteria, making them strong candidates for repurposing against DEPDC1. To better understand the molecular basis of these interactions, docking poses of the three selected ligands were visualized and analyzed in (Figure 2) as shown in Figure 2a–c, all three compounds successfully occupy the same binding pocket of the DEPDC1 protein, interacting with crucial residues such as TRP, ARG, ASN, and PHE. Delafloxacin forms stabilizing hydrogen bonds and hydrophobic interactions within the cavity, particularly with PHE and ASN, at a bond distance of approximately 3.7 Å. Riboflavin and Afatinib similarly exhibit hydrogen bonding interactions with residues such as TRP, THR, and ARG, consistent with their docking scores. The binding site overview in (Figure 2d) further illustrates the shared binding pocket and key interacting residues, while the 2D chemical structures in (Figure 2e) highlight the structural diversity of the ligands. These visualizations confirm that Delafloxacin, Riboflavin, and Afatinib exhibit spatial and chemical compatibility with the DEP domain, reinforcing the validity of the docking predictions and justifying their selection for downstream experimental evaluation.

**FIGURE 2:**
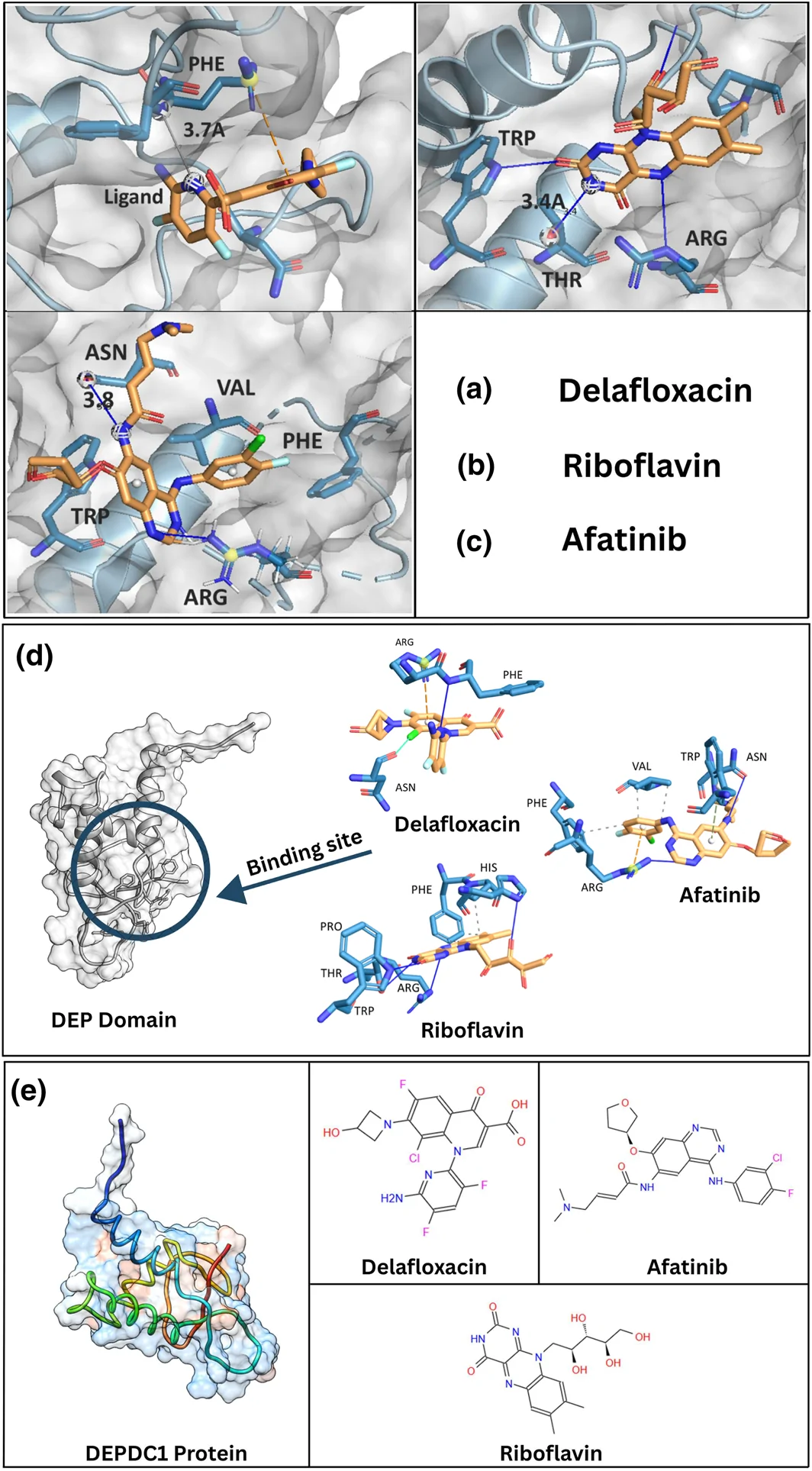
Binding interaction of protein and drugs. (a–c) Binding poses of Delafloxacin, Riboflavin, and Afatinib showing the key interactions with residues. (d) Binding site overview highlighting shared contact residues stabilizing ligand interactions. (e) 2D structures of the ligands and the DEPDC1 protein surface model, demonstrating structural compatibility and ligand diversity.

### 3.4 Drug‐likeness assessment using Lipinski's rule of five

The pharmacokinetic suitability of those top‐ranked compounds was evaluated using Lipinski's Rule of Five (RO5), which considers key properties associated with oral bioavailability, including molecular weight ≤ 500 Da, no more than 5 hydrogen bond donors, no more than 10 hydrogen bond acceptors, and a logP ≤ 5. The analysis revealed that Delafloxacin and Afatinib met these criteria. Delafloxacin and Riboflavin, fluoroquinolone antibiotics, exhibited strong binding affinities (−7.1 and −7.2 kcal/mol, respectively), but Delafloxacin showed more favorable drug‐like characteristics, highlighting its potential for repurposing. Afatinib, an EGFR inhibitor used in oncology, also demonstrated a high binding affinity (−6.8 kcal/mol) and met all RO5 parameters, indicating promise for alternative therapeutic applications (Table 3).

**TABLE 3:** Molinspiration properties scores of DEPDC1 for ADMET analysis.

Chemical	miLogP	TPSA	Molecular weight	H‐bond acceptor	H‐bond donors	No. of rotatable bonds	No. of violations
ZINC000003827556	−0.70	121.69	440.76	8	4	3	0
ZINC000003830944	−2.99	199.87	821.14	12	8	12	3
ZINC000002539702	−2.11	107.22	178.14	6	4	1	0
ZINC000001883067	1.45	157.70	229.10	10	1	3	0
ZINC000003813061	3.45	118.72	364.42	7	4	8	0
ZINC000000020220	−0.70	74.57	331.35	6	2	3	0
ZINC000000592419	4.26	60.46	403.21	5	1	7	0
ZINC000003918138	−3.51	198.22	332.31	11	9	7	2
ZINC000003976838	4.21	88.61	485.95	8	2	8	0
ZINC000001530922	1.11	116.17	362.28	8	3	5	0
ZINC000003842753	−0.99	104.56	296.20	7	3	3	0
ZINC000003826253	0.39	83.80	401.44	7	2	4	0
ZINC000004038341	2.06	102.25	424.99	7	4	7	0
ZINC000002036848	−0.76	161.56	376.37	10	5	5	0
ZINC000001035331	−2.77	143.73	244.21	9	5	3	0

Among the top candidates, Delafloxacin and Afatinib satisfied all Lipinski criteria, indicating favorable oral bioavailability potential. Riboflavin, despite having the highest binding affinity (−7.2 kcal/mol), exhibited higher polar surface area and hydrogen‐bond donor/acceptor counts, which may limit membrane permeability. Delafloxacin's combination of strong binding affinity (−7.1 kcal/mol) and favorable drug‐likeness supported its selection for subsequent experimental validation.

### 3.5 In vitro growth inhibition of lung cancer cells

The in vitro growth inhibition data demonstrate that all three tested compounds, Delafloxacin, Riboflavin, and Afatinib, exert inhibitory effects on lung cancer cell proliferation in a dose‐dependent manner. Among them, Delafloxacin exhibited the most potent anti‐proliferative activity, reducing cell viability to below 10% at the highest tested concentration (128 μg/mL), indicating strong cytotoxic potential against NCI‐H1299 lung cancer cells. Riboflavin also showed a strong inhibitory effect, reducing cell proliferation to approximately 30% at the same concentration, whereas Afatinib was comparatively less effective, achieving about 40% inhibition. These results suggest that Delafloxacin is the most effective compound in suppressing lung cancer cell growth in vitro, followed by Riboflavin, with Afatinib showing moderate activity. This trend supports the molecular docking findings and highlights Delafloxacin as the most promising candidate for further preclinical evaluation (Figure 3).

**FIGURE 3:**
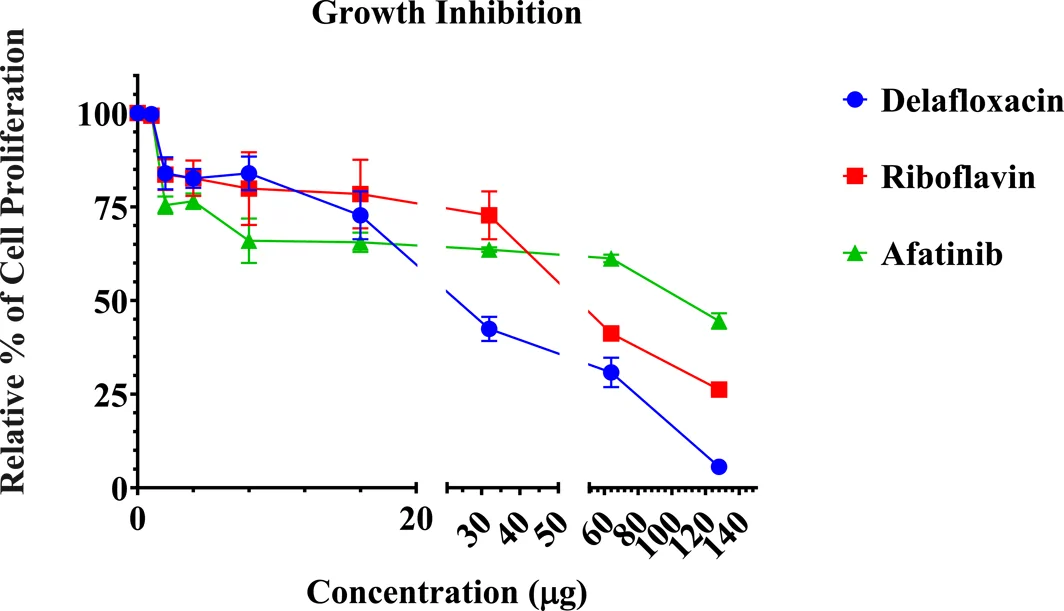
Dose‐dependent inhibition of NCI‐H1299 cell proliferation by Delafloxacin, Riboflavin, and Afatinib. Cells were treated with increasing concentrations (0–128 μg/mL) of the indicated compounds for 72 h, and cell viability was assessed using a proliferation assay. Delafloxacin showed the most potent concentration‐dependent growth inhibition with nearly complete suppression at higher doses. Riboflavin and Afatinib also reduced proliferation though to a lesser extent. Data represent mean ± SEM from at least three independent experiments.

### 3.6 Delafloxacin downregulates DEPDC1 and the downstream signaling pathway

Quantitative real‐time PCR analysis revealed that treatment with delafloxacin at concentrations of 10, 20, and 40 μg/mL resulted in a robust, dose‐dependent downregulation of evaluated genes compared to the untreated control group (Figure 4). These genes DEPDC1, RAS, EGFR, KIF2C, CDK1, CCNB2, MAPK1, NID1, FN1, BIRC5, and TNFSF12 are known to play crucial roles in cancer‐related signaling pathways, cell cycle progression, apoptosis inhibition, and extracellular matrix remodeling. Among them, DEPDC1, KIF2C, and CDK1 exhibited the most dramatic reductions, with relative expression levels declining from approximately 6.5‐, 6.0‐, and 5.5‐fold in the control group to about 1.5‐, 1.3‐, and 1.2‐fold, respectively, at 40 μg/mL. This trend was consistent across all genes examined, with significant differences observed at every concentration tested (*p* < 0.0001).

**FIGURE 4:**
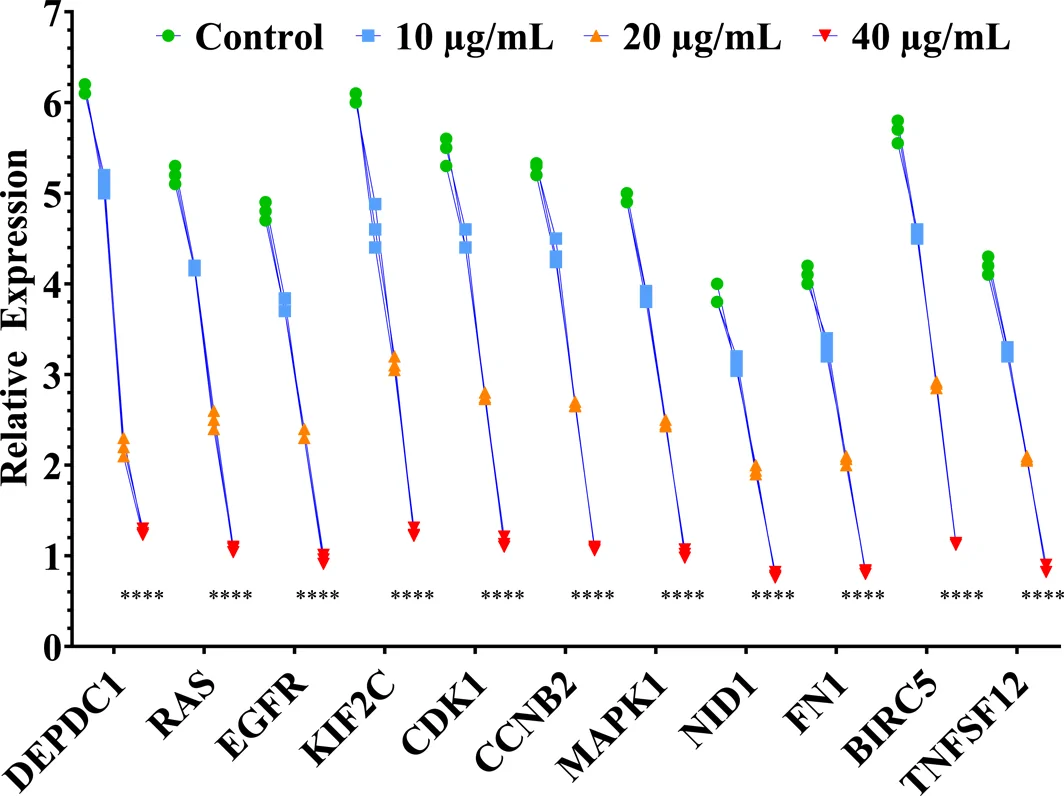
RT‐qPCR analysis: Delafloxacin treatment is associated with downregulation of DEPDC1 and downstream genes. Cells were treated with 10, 20, and 40 μg/mL Delafloxacin for 24 h, and RT‐qPCR quantified relative gene expression. Genes involved in proliferation (DEPDC1, RAS, EGFR), mitotic regulation (Cyclin‐dependent kinase 1 (CDK1), CCNB2, KIF2C), survival (BIRC5), signaling (MAPK1, TNFSF12), and extracellular matrix remodeling (FN1, NID1) showed dose‐dependent downregulation. Data are shown as fold changes relative to control, with technical replicates plotted. Treatment led to significant suppression of G2/M regulators (CDK1, CCNB2), suggesting impaired mitotic entry.

Genes involved in cell proliferation signaling, such as RAS and EGFR, showed notable suppression at 10 μg/mL, suggesting that the compound may act upstream in growth factor‐mediated pathways. Additionally, CCNB2 and MAPK1, both key regulators of the cell cycle, were significantly downregulated, reflecting a potential blockade at critical cell cycle checkpoints. Genes associated with extracellular matrix integrity and remodeling, such as FN1 and NID1, were also reduced, indicating a possible impairment in cellular adhesion and invasion mechanisms. BIRC5, which encodes Survivin, a known inhibitor of apoptosis and a marker of poor prognosis in several cancers, was profoundly suppressed in a dose‐dependent fashion, as was TNFSF12, a cytokine involved in inflammatory signaling and tumor immune evasion.

The observed gene suppression in response to compound treatment provides compelling evidence of its multifaceted anti‐cancer activity. The consistent, dose‐dependent downregulation of genes such as CDK1, CCNB2, and MAPK1 suggests a potential mechanism involving disruption of the G2/M phase transition and mitotic progression, which are critical for unchecked cancer cell proliferation. Cyclin‐dependent kinase 1, in particular, is a master regulator of mitosis, and its downregulation may lead to mitotic catastrophe or senescence, thereby limiting tumor expansion. Similarly, KIF2C and DEPDC1, which are involved in mitotic spindle dynamics and chromosomal segregation, respectively, were significantly repressed, indicating a likely disruption in mitotic fidelity and cell division.

Furthermore, suppression of EGFR and RAS, two prominent upstream regulators of proliferative signaling, suggests that the compound may inhibit oncogenic signaling cascades such as the MAPK/ERK and PI3K/AKT pathways, both of which are commonly hyperactivated in solid tumors. Inhibition of FN1 and NID1 suggests that the compound may impair tumor cell adhesion, migration, and metastatic potential by interfering with extracellular matrix interactions. The marked reduction of BIRC5 (Survivin) aligns with pro‐apoptotic outcomes, as BIRC5 plays a pivotal role in promoting cell survival and resistance to chemotherapy. Notably, downregulation of TNFSF12, a gene associated with immune modulation and tumor progression, may indicate a dual anti‐proliferative and immunomodulatory effect of the compound.

Taken together, these findings underscore delafloxacin's ability to simultaneously target multiple oncogenic pathways, cell cycle regulators, and metastatic markers. While these transcriptional changes are consistent with anti‐proliferative activity, they represent correlative findings. Whether DEPDC1 downregulation is a direct consequence of Delafloxacin treatment or an indirect effect of cell cycle perturbation cannot be determined from these data alone.

### 3.7 DEPDC1 inhibition arrests cells in the G2/M phase

Flow cytometric analysis revealed that Delafloxacin induces a concentration‐dependent G2/M arrest in NCI‐H1299 cells. In untreated control cells, the distribution of cell cycle phases showed 42.86% in G1, 42.03% in S, and 12.00% in G2/M. Upon treatment with 10 μg/mL Delafloxacin, the G2/M population increased to 17.61%, with a reduction in S‐phase cells (26.45%). Notably, at 20 μg/mL, G2/M phase accumulation remained elevated at 32.43%, while S‐phase cells decreased to 23.17%. A corresponding increase in sub‐G1 populations (15.53% at 10 μg/mL and 1.97% at 20 μg/mL) suggests potential apoptotic induction at lower doses described in (Figure 5).

**FIGURE 5:**
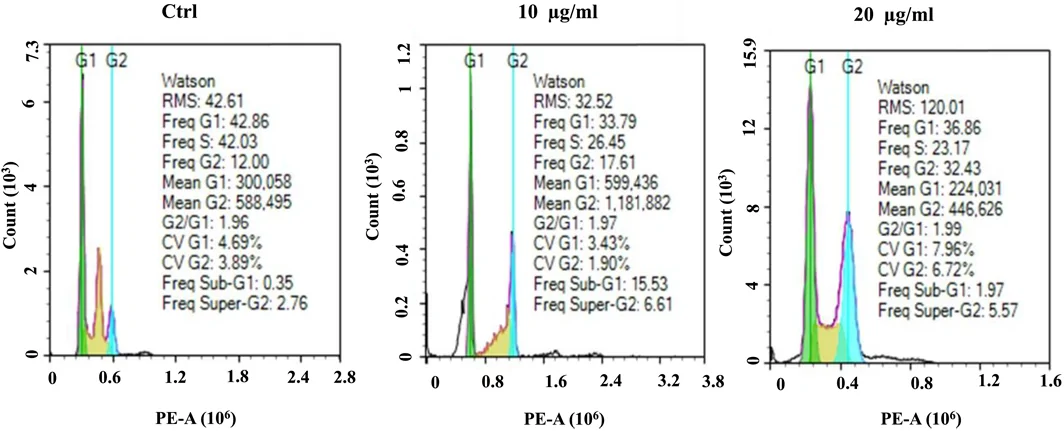
Delafloxacin induces G2/M phase arrest in NCI‐H1299 cells. Flow cytometry‐based cell cycle analysis was performed after 24 h treatment with 0 (Ctrl), 10, or 20 μg/mL Delafloxacin. DNA content was stained with propidium iodide (PI) and analyzed using Watson's model fitting. Delafloxacin‐treated cells showed a concentration‐dependent increase in the G2/M population and a decrease in S‐phase cells, indicative of G2/M arrest. Sub‐G1 populations were also elevated at 10 μg/mL, suggesting early apoptosis. These findings corroborate RT‐qPCR results showing reduced expression of mitotic regulators.

This G2/M arrest phenotype aligns with gene expression data showing significant downregulation of CDK1 and CCNB2, two key regulators of mitotic entry. Cyclin‐dependent kinase 1 partners with cyclin B2 (encoded by *CCNB2*) to form the maturation‐promoting factor (MPF), which is essential for the G2 to M transition. Suppressing both genes likely compromises MPF complex activity, halting cell cycle progression at the G2/M checkpoint. This mechanistic link underscores the functional impact of DEPDC1 suppression and its downstream gene network during Delafloxacin treatment.

The transcriptional downregulation of CDK1 and CCNB2 observed by RT‐qPCR is consistent with impaired formation of the cyclin B‐CDK1 complex, the master regulator of the G2/M transition. Reduced expression of these genes would be expected to delay or prevent mitotic entry, favoring G2 arrest rather than mitotic catastrophe. This interpretation aligns with the observed G2/M accumulation but requires confirmation at the protein and functional levels.

### 3.8 In vivo anti‐tumor efficacy of delafloxacin

Animal testing was conducted over 42 days to assess the therapeutic potential of these compounds in vivo. The anti‐tumor efficacy of Delafloxacin was further validated in vivo using a lung cancer xenograft mouse model over a 42‐day treatment period. As illustrated in (Figure 6), Delafloxacin‐treated mice exhibited a marked reduction in tumor volume compared to the control group, with statistically significant differences observed from day 18 onwards (*p* < 0.001). At the end of the treatment, tumor volume in the Delafloxacin group remained consistently suppressed, whereas tumors in the control group continued to grow rapidly. Furthermore, the final tumor weight analysis showed a significant decrease in the Delafloxacin group (*p* < 0.0001), confirming the compound's potent inhibitory effect on tumor growth. Significantly, Delafloxacin treatment did not adversely affect the overall health status of the mice, as evidenced by stable body weights throughout the study period, comparable to the normal (non‐tumor‐bearing) group. This indicates that Delafloxacin effectively inhibits lung cancer progression in vivo while maintaining a favorable safety profile, underscoring its potential for further development as an anti‐cancer therapeutic. The treatments demonstrated promising inhibitory effects on tumor progression, supporting the docking predictions and highlighting their potential as repurposed therapeutics for lung cancer.

**FIGURE 6:**
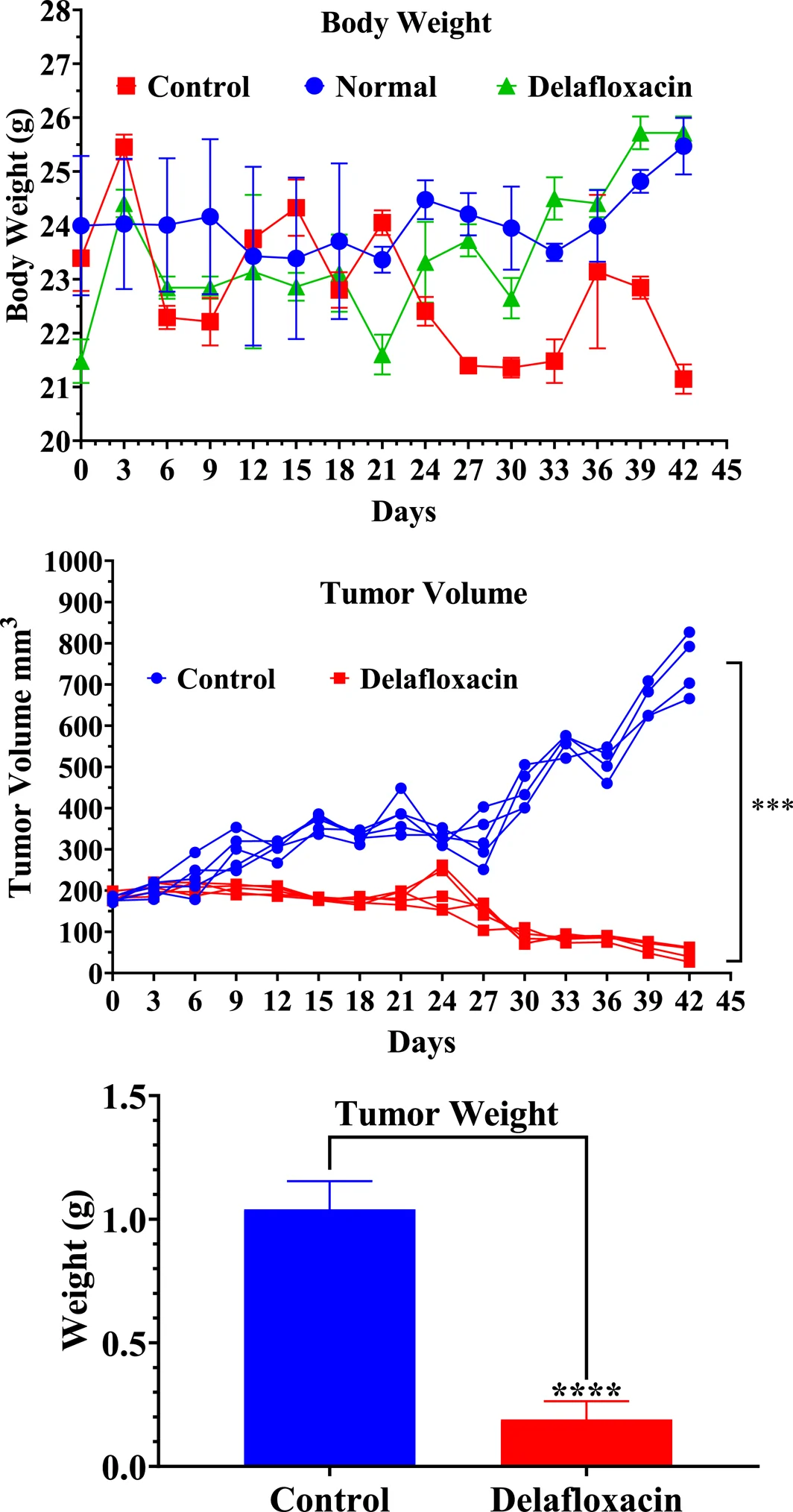
In vivo evaluation of Delafloxacin effects in Balb/C mice model. Mice xenograft models of NCI‐H1299 cells were treated with Delafloxacin via intravenous injection for 42 days. The results showed improved body weight and decreased tumor volume and weight compared with the control group.

Measurement of tumor size is essential in preclinical animal studies when assessing responses to cancer treatment. Sequential tumor volume measurements with a non‐invasive method are crucial in longitudinal studies. The current standard technique for measuring subcutaneous tumor xenografts is by caliper, where tumor volume is calculated as (W2 x L)/2. In this study, we demonstrate that Delafloxacin treatment exhibited an anti‐tumorogenic effect. In addition, Delafloxacin showed anti‐cancer activity in vitro and in vivo against chemo‐resistant cancers. In our studies, the Delafloxacin‐treated mice did not lose weight and did not show any signs of suffering from drug toxicity.

Furthermore, Delafloxacin showed potent anti‐tumor activity without causing significant body weight loss in the treated mouse xenograft model. Therefore, Delafloxacin may be a promising drug candidate. Our goal is to revolutionize lung cancer treatment regimens by replacing them with more effective and less toxic regimens. While the xenograft study demonstrated significant tumor growth inhibition and tolerability of Delafloxacin treatment, tumor tissues were not subjected to pharmacodynamic analysis. Therefore, we did not assess DEPDC1 protein expression, phospho‐CDK1 status, Cyclin B1 levels, proliferation markers (e.g., Ki67), or apoptosis markers (e.g., cleaved caspase‐3) within the excised tumors. Consequently, although the in vivo efficacy is consistent with the in vitro anti‐proliferative and G2/M arrest phenotype, we cannot directly confirm that tumor suppression was mediated through DEPDC1‐associated molecular mechanisms in vivo. Thus, the mechanistic linkage between the in vitro molecular findings and the in vivo anti‐tumor effect remains inferential. Future studies incorporating tumor tissue analysis will be required to validate target engagement and pathway modulation in vivo.

## 4 CONCLUSION AND FUTURE PERSPECTIVES

This study identifies Delafloxacin, an FDA‐approved antibiotic, as a candidate for repurposing in DEPDC1‐overexpressing lung cancer. Through integrated computational screening, in vitro assays, and in vivo validation, we demonstrate that Delafloxacin treatment suppresses DEPDC1 and downstream regulators of proliferation and mitotic progression (CDK1, CCNB2, KIF2C), leading to G2/M phase accumulation and reduced tumor growth in a xenograft model. These findings provide the first evidence that a fluoroquinolone antibiotic may exert anti‐cancer activity in NSCLC cells through mechanisms correlating with DEPDC1 downregulation.

The strengths of this study include systematic screening of 1399 FDA‐approved compounds, integration of computational predictions with experimental validation, and demonstration of in vivo efficacy with a favorable safety profile. The differential binding affinities observed among structurally related fluoroquinolones (Delafloxacin −7.1 kcal/mol; moxifloxacin −6.8 kcal/mol; ciprofloxacin −6.7 kcal/mol) suggest structure–activity relationship features that may inform future medicinal chemistry optimization. Furthermore, the dose‐dependent transcriptional suppression of multiple oncogenic regulators (RAS, EGFR, CDK1, CCNB2, BIRC5) indicates that Delafloxacin's effects extend beyond a single pathway, potentially offering therapeutic advantage in tumors with complex driver landscapes.

Several considerations should inform the interpretation of these results and guide future investigations. First, the observed DEPDC1 downregulation represents a correlative finding rather than validated direct targeting. Molecular docking predicts a favorable binding interaction (binding energy −7.1 kcal/mol); however, computational predictions alone do not constitute target validation. Future studies employing DEPDC1 knockdown and overexpression models are necessary to establish causality, and biophysical methods such as surface plasmon resonance or cellular thermal shift assay are required to confirm direct binding. Second, while transcriptional downregulation of CDK1 and CCNB2 was observed, protein‐level validation (western blot for CDK1, Cyclin B1, phospho‐CDK1) and functional assessment of mitotic entry (phospho‐histone H3 staining) are needed to determine whether cells arrest in G2 versus M phase. Third, the IC_50_ of approximately 30 μg/mL (∼68 μM) is consistent with reported concentrations for repurposed antibiotics but exceeds clinically achievable peak serum concentrations (8–10 μg/mL). This suggests that while Delafloxacin itself may not be directly translatable at standard dosing, it provides a validated chemical scaffold for potency optimization or investigation in localized delivery contexts. Fourth, the generalizability of these findings across NSCLC subtypes remains to be established, as experiments were conducted exclusively in NCI‐H1299 cells. Validation in additional lines representing LUAD (A549, PC9) and squamous cell carcinoma is warranted. Fifth, the in vivo study lacked pharmacodynamic endpoints; future xenograft studies should incorporate tumor harvests for IHC analysis of DEPDC1, Ki67, and cleaved caspase‐3 to confirm the mechanism of action in vivo.

Despite these considerations, this work provides a foundation for multiple lines of investigation. The differential activity among fluoroquinolones suggests that structure–activity relationship studies could yield analogs with improved potency. The association between DEPDC1 suppression and anti‐tumor activity positions DEPDC1 as a potential biomarker for patient selection in future trials. The favorable safety profile observed in vivo (stable body weight, no overt toxicity) supports further preclinical development.

In conclusion, this study positions Delafloxacin as a candidate for repurposing in DEPDC1‐associated malignancies while transparently defining the evidence gaps that must be addressed in subsequent investigations. The integration of computational screening with experimental validation demonstrates the power of drug repurposing approaches to identify unexpected anti‐cancer activities in approved drugs. Whether Delafloxacin itself, a structurally optimized derivative, or a combination strategy ultimately proves clinically useful, the present findings contribute to the growing recognition that antibiotic scaffolds may harbor previously unrecognized anti‐cancer properties worthy of systematic investigation.

## AUTHOR CONTRIBUTIONS

**Noman Ali:** Conceptualization; methodology; investigation; writing—original draft. **Farishta Shafiq:** Methodology; data curation. **Mishal Iftikar:** Methodology; data curation. **Muhammad Shahzad Zafar:** Methodology; data curation; data collection. **Muhammad Shoaib:** Conceptualization; methodology; formal analysis, data curation.

## CONFLICT OF INTEREST STATEMENT

The authors declare no conflicts of interest.

## ETHICS STATEMENT

Ethical Approval for Animal Studies: All animal experiments were conducted in accordance with the ethical guidelines of the Institute of Precision Diagnosis and Treatment of Digestive System Tumors, Shenzhen University. The study was approved by the IACUC. Mice were housed under SPF conditions with ad libitum access to food and water. All procedures were performed to minimize animal suffering and in compliance with international ethical standards for animal research. Ethical Approval for Cell Culture: The NCI‐H1299 lung cancer cell line used in this study was obtained from a commercially available source (ATCC) and was handled in accordance with institutional and ethical standards for the use of human‐derived cell lines in research. No patient‐specific data were used in this study.

## Data Availability

The data that support the findings of this study are available on request from the corresponding author. The data are not publicly available due to privacy or ethical restrictions.

## References

[1] D. A. Singh , PEXACY Int. J. Pharm. Sci. 2023, 2, 59.

[2] T. Lu , X. Yang , Y. Huang , M. Zhao , M. Li , K. Ma , J. Yin , C. Zhan , Q. Wang , Cancer Manag. Res. 2019, 11, 943. 10.2147/CMAR.S187317 PMC6345192 30718965

[3] M. Dowling , N. Efstathiou , A. Drury , C. Semple , P. Fernández‐Ortega , K. Brochstedt Dieperink , E. Pape , G. Kotronoulas , S. Miguel , S. Colomer‐Lahiguera , G. Bağçivan , Eur. J. Oncol. Nurs. 2023, 63, 102272. 10.1016/j.ejon.2023.102272 36827837

[4] R. L. Siegel , K. D. Miller , A. Jemal , CA Cancer J. Clin. 2018, 68, 7. 10.3322/caac.21442 29313949

[5] P. Chasta , A Review Article on Lung Cancer for Non‐Smokers Recent Trend 2021.

[6] D. Liu , H. Li , J. Ouyang , Oncol. Lett. 2024, 28, 1.

[7] Y. Yang , Y. Jiang , M. Jiang , J. Zhang , B. Yang , Y. She , W. Wang , Y. Deng , Y. Ye , Exp. Mol. Pathol. 2016, 100, 344. 10.1016/j.yexmp.2016.03.002 26970279

[8] S. G. Yuan , W. J. Liao , J. J. Yang , G. J. Huang , Z. Q. Huang , J. Cancer Prev. 2014, 15, 10917. 10.7314/apjcp.2014.15.24.10917 25605201

[9] M. Kanehira , Y. Harada , R. Takata , T. Shuin , T. Miki , T. Fujioka , Y. Nakamura , T. Katagiri , Oncogene 2007, 26, 6448. 10.1038/sj.onc.1210466 17452976

[10] L. Zhang , Y. Du , S. Xu , Y. Jiang , C. Yuan , L. Zhou , X. Ma , Y. Bai , J. Lu , J. Ma , Cancer Lett. 2019, 442, 242. 10.1016/j.canlet.2018.11.003 30419349

[11] L. Huang , K. Chen , Z. peng Cai , F. chao Chen , H. yong Shen , W. hua Zhao , S. jie Yang , X. biao Chen , G. xue Tang , X. Lin , Biochem. Biophys. Res. Commun. 2017, 490, 707. 10.1016/j.bbrc.2017.06.105 28634077

[12] W. Guo , H. Li , H. Liu , X. Ma , S. Yang , Z. Wang , Oncol. Rep. 2019, 42, 1075. 10.3892/or.2019.7221 PMC6667871 31322256

[13] B. Jia , J. Liu , X. Hu , L. Xia , Y. Han , Ann. Transl. Med. 2022, 10, 1355. 10.21037/atm-22-5598 PMC9843344 36660720

[14] H. Liao , J. Zheng , J. Lu , H. L. Shen , Mol. Neurobiol. 2024, 62662, 6998. 10.1007/s12035-024-04634-2 39560902

[15] L. Wang , Y. Xue , X. Wang , Y. Pan , S. Li , J. Mei , S. Jiang , Q. Zheng , Y. Liu , Y. Liu , J. Yuan , Y. Ma , Nano Today 2024, 56, 102249.

[16] M. Lv , X. Li , Z. Yin , H. Yang , B. Zhou , PLoS One 2024, 19, 1. 10.1371/journal.pone.0294227 PMC10986975 38564630

[17] T. Lengauer , M. Rarey , Curr. Opin. Struct. Biol. 1996, 6, 402. 10.1016/s0959-440x(96)80061-3 8804827

[18] Y. Zhao , Q. Liu , J. Du , Q. Meng , L. Zhang , Smart Mol. 2023, 1, e20230012. 10.1002/smo.20230012 PMC12118191 40626207

[19] Z. Zhao , X. Tong , J. Sun , J. Lu , G. Zhang , Q. Wang , L. Yang , Smart Mol. 2025, 3, e20250002. 10.1002/smo.20250002 PMC12483134 41035515

[20] S. Tang , J. Ding , X. Zhu , Z. Wang , H. Zhao , J. Wu , bioRxiv 2023, 14.

[21] T. Nakane , A. Kotecha , A. Sente , G. Mcmullan , P. M. G. E. Brown , I. T. Grigoras , L. Malinauskaite , J. Miehling , T. Uchański , L. Yu , D. Karia , V. Pechnikova , E. De Jong , J. Keizer , M. Bischoff , J. Mccormack , P. Tiemeijer , S. W. Hardwick , D. Y. Chirgadze , G. Murshudov , A. Radu , S. H. W. Scheres , EM At. resolution 2021, 587, 152. 10.1038/s41586-020-2829-0 PMC7611073 33087931

[22] P. C. Agu , C. A. Afiukwa , O. U. Orji , E. M. Ezeh , I. H. Ofoke , C. O. Ogbu , E. I. Ugwuja , P. M. Aja , Sci. Rep. 2023, 13, 1. 10.1038/s41598-023-40160-2 PMC10435576 37592012

[23] A. V. Sadybekov , V. Katritch , Nat 2023, 673. 10.1038/s41586-023-05905-z 37100941

[24] P. P. Parvatikar , S. Patil , K. Khaparkhuntikar , S. Patil , P. K. Singh , R. Sahana , R. V. Kulkarni , A. V. Raghu , Antivir. Res 2023, 220, 105740. 10.1016/j.antiviral.2023.105740 37935248

[25] H. Li , Y. Liu , H. Zhang , X. Wang , Oncol. Lett. 2025, 29, 1.

[26] Z. Gong , H. Chu , J. Chen , L. Jiang , B. Gong , P. Zhu , C. Zhang , Z. Wang , W. Zhang , J. Wang , C. Li , H. Zhao , Cancer Biomarkers 2021, 30, 299. 10.3233/CBM-201760 PMC12499972 33361586

[27] J. Gupta , B. I. Saeed , A. K. Bishoyi , A. G. Alkhathami , S. Asliddin , D. Nathiya , M. Ravi Kumar , D. Bhanot , A. B. Rashed , Y. F. Mustafa , Med. Oncol. 2025, 42942, 422. 10.1007/s12032-025-02973-1 40782258

[28] M. Hu , P. Tao , Y. Wang , C. Zhu , Y. Ma , X. Liu , H. Cai , Sci. Rep. 2025, 15115, 5703. 10.1038/s41598-025-89948-4 PMC11832750 39962178

[29] R. Q. Li , Y. Yang , L. Qiao , L. Yang , D. D. Shen , X. J. Zhao , Biomed. Pharmacother. 2024, 171, 116173. 10.1016/j.biopha.2024.116173 38237349

[30] X. Feng , C. Zhang , L. Zhu , L. Zhang , H. Li , L. He , Y. Mi , Y. Wang , J. Zhu , Y. Bu , Oncotarget 2017, 8, 63605. 10.18632/oncotarget.18868 PMC5609947 28969015

[31] O. Trott , A. J. Olson , J. Comput. Chem. 2010, 31, 455. 10.1002/jcc.21334 PMC3041641 19499576

